# Supplementary material for: Quantitative Structure–Electrochemistry Relationship (QSER) Studies on Metal–Amino–Porphyrins for the Rational Design of CO2 Reduction Catalysts
Source: Molecules. 2023 Mar 30;28(7):3105. doi: 10.3390/molecules28073105 (PMC10096077; doi:10.3390/molecules28073105)
Supplement: Supplementary file 1 [file molecules-28-03105-s001.zip › molecules-2299533-supplementary.docx]

**Supplementary Materials**

Quantitative Structure–electrochemistry Relationship (QSER) Studies on Metal-Amino-Porphyrins for Rational Design of CO_2_ Reduction Catalysts

| **No** | Figure/Table | Page |
| --- | --- | --- |
| 1. | Table S1. The obtained QSER equations to predict the Gibbs free energy values of H* (Y1) for TM-PPs. | S3 |
| 2. | **Table S2**. The obtained QSER equations to predict the Gibbs free energy values of C*OOH (Y2) for TM-PPs. | S4 |
| 3. | Table S3. The obtained QSER equations to predict the Gibbs free energy values of O*CHO (Y3) for TM-PPs. | S5 |
| 4. | **Table S4**. The predicted Gibbs free energy values of H*, C*OOH and O*CHO by QSER models for TM-Amino-TPPs. | S6 |
| 5. | **Table S5**. The selected 3 properties values of newly designed transitional metal porphyrin complexes. | S7 |
| 6. | **Figure S1**. Occurrences of populations of 8 properties for (a) G(H*), (b) G(C*OOH), (c) G(O*CHO) prediction by genetic algorithm (GA) method. | S8 |
| 7. | **Figure S2**. The plot of the descriptors (a) X1, (b) X3 and (c) X7 values of TM-PPs versus TM-Amino-TPPs. | S8 |

**Table S1**. The obtained QSER equations to predict the Gibbs free energy values of H* (Y1) for TM-PPs.

| **No** | **Equation** | **F-value** | **R^2^** | **R^2^(CV)** | **RSS** | **RMSE** |
| --- | --- | --- | --- | --- | --- | --- |
| Eq. 1 | Y1 = - 5.994 * X1- 14.18 * X3+ 35.934 * X7 - 60.834 | 9.089 | 0.820 | 0.603 | 0.754 | 0.275 |
| Eq. 1A | Y1 = 383.311 * ramp (X3 - 0.183)  + 29.037 * ramp (X7 - 1.953)  + 33.1439 * ramp (2.069 - X7)  - 3.440 | 34.273 | 0.945 | 0.832 | 0.231 | 0.152 |
| Eq. 1B | Y1 = 10.271 * ramp (X1 - 1.019)  + 401.503 * ramp(X3 - 0.184)  + 11.016 * ramp( 1.733 - X1)  - 7.352 | 32.113 | 0.941 | 0.804 | 0.245 | 0.157 |
| Eq. 1C | Y1 = 381.573 * ramp (X3 - 0.183)  + 35.183 * ramp (X7 - 1.949)  + 39.346 * ramp (2.075 - X7)  - 4.532 | 34.286 | 0.945 | 0.835 | 0.230 | 0.152 |
| Eq.1D | Y1 = 16.1652 * ramp (X7 - 1.957)  + 3.189 * ramp (1.706 - X1)  - 1.345 | 3.029 | 0.464 | 0.000 | 2.241 | 0.871 |

**Table S2**. The obtained QSER equations to predict the Gibbs free energy values of C*OOH (Y2) for TM-PPs.

| **No** | **Equation** | **F-value** | **R^2^** | **R^2^(CV)** | **RSS** | **RMSE** |
| --- | --- | --- | --- | --- | --- | --- |
| Eq. 2 | Y2 = - 5.850 * X1 - 17.701* X3 + 33.585 * X7- 56.143 | 12.238 | 0.860 | 0.683 | 0.471 | 0.217 |
| Eq. 2A | Y2 = 11.025* ramp (X1 - 1.011)  + 367.618 * ramp(X3 - 0.184)  + 11.838 * ramp( 1.768 - X1)  - 8.726 | 44.384 | 0.957 | 0.924 | 0.144 | 0.120 |
| Eq. 2B | Y2 = 10.271 * ramp (X1 - 1.019)  + 401.503 * ramp(X3 - 0.184)  + 11.016 * ramp( 1.733 - X1)  - 7.352 | 44.372 | 0.957 | 0.923 | 0.145 | 0.1120 |
| Eq. 2C | Y2 = 332.971 * ramp (X3 - 0.183)  + 41.718 * ramp (X7 - 1.946)  + 46.059 * ramp (2.087 - X7)  - 6.345 | 38.441 | 0.951 | 0.916 | 0.166 | 0.129 |
| Eq. 2D | Y2 = 14.860 * ramp (X7 - 1.957)  + 3.002 * ramp (1.799 - X1)  - 1.851 | 3.856 | 0.524 | 0.047 | 1.594 | 0.527 |

**Table S3**. The obtained QSER equations to predict the Gibbs free energy values of O*CHO (Y3) for TM-PPs.

| **No** | **Equation** | **F-value** | **R^2^** | **R^2^(CV)** | **RSS** | **RMSE** |
| --- | --- | --- | --- | --- | --- | --- |
| Eq. 3 | Y3 = - 4.122 * X1 + 11.431 * X7 - 17.236 | 9.280 | 0.726 | 0.574 | 1.692 | 0.404 |
| Eq. 3A | Y3 = 2354.560 * ramp (X3 - 0.177)  - 2043.245 * ramp (X3 - 0.176)  + 25.754 * ramp (2.033 - X7)  - 1.010 | 42.490 | 0.955 | 0.907 | 0.278 | 0.167 |
| Eq. 3B | Y3 = 244.003 * ramp (X3 - 0.184)  + 2.692 * ramp( 1.635 - X1)  - 1.031 | 14.212 | 0.802 | 0.670 | 1.221 | 0.349 |
| Eq. 3C | Y3 = - 832.681 * ramp (X3 - 0.176)  + 1141.851 * ramp (X3 - 0.178)  + 25.710 * ramp (2.033 - X7)  - 1.009 | 42.476 | 0.955 | 0.907 | 0.278 | 0.167 |
| Eq. 3D | Y3 =- 25.354* ramp (X1 - 1.433)  + 31.147 * ramp (X1 - 1.547)  + 0.438 | 9.735 | 0.736 | 0.641 | 1.634 | 0.483 |

**Table S4**. The predicted Gibbs free energy values of H*, C*OOH and O*CHO by QSER models for TM-Amino-TPPs.

| **TM-Amino-TPPs** | **Predicted G(H*) values/eV** | | **Predicted G(C*OOH) values/eV** | | **Predicted G(O*CHO) values/eV** | |
| --- | --- | --- | --- | --- | --- | --- |
|  | **Eq. 1** | **Eq. 1A** | **Eq. 2** | **Eq. 2A** | **Eq. 3** | **Eq. 3A** |
| Sc | 1.466 | 1.075 | 0.869 | 0.395 | -0.72 | -1.01 |
| Ti | -0.18 | 0.066 | -0.611 | -0.373 | -1.068 | -1.01 |
| V | 0.56 | 0.14 | 0.099 | -0.166 | -0.222 | -0.812 |
| Cr | 2.191 | 0.459 | 1.658 | 0.1 | 1.068 | -0.264 |
| Mn | 0.376 | 0.271 | -0.04 | -0.031 | 0.096 | 0.012 |
| Fe | -0.09 | 0.328 | -0.463 | -0.032 | -0.068 | 0.373 |
| Co | 0.229 | 0.419 | -0.138 | 0.128 | 0.493 | -0.15 |
| Ni | 0.233 | 1.15 | -0.11 | 0.834 | 0.88 | 1.094 |
| Cu | 1.519 | 1.687 | 1.017 | 1.179 | 0.892 | 1.159 |
| Zn | 1.719 | 1.918 | 1.154 | 1.395 | 0.549 | 0.654 |

**Table S5**. The selected 3 properties values of newly designed transitional metal porphyrin complexes.

| **TM-Amino-TPP** | **X1**  **NBO charges of TM** | **X3**  **The average NBO charges of C_a_** | **X7**  **The average bond length of TM-N_a_ and TM-N_b_** |
| --- | --- | --- | --- |
| Sc | 1.839 | 0.171 | 2.108 |
| Ti | 1.743 | 0.163 | 2.043 |
| V | 1.488 | 0.173 | 2.025 |
| Cr | 1.161 | 0.184 | 2.020 |
| Mn | 1.322 | 0.175 | 1.993 |
| Fe | 1.323 | 0.172 | 1.979 |
| Co | 1.126 | 0.177 | 1.957 |
| Ni | 0.960 | 0.181 | 1.931 |
| Cu | 1.151 | 0.187 | 2.001 |
| Zn | 1.337 | 0.188 | 2.038 |

**
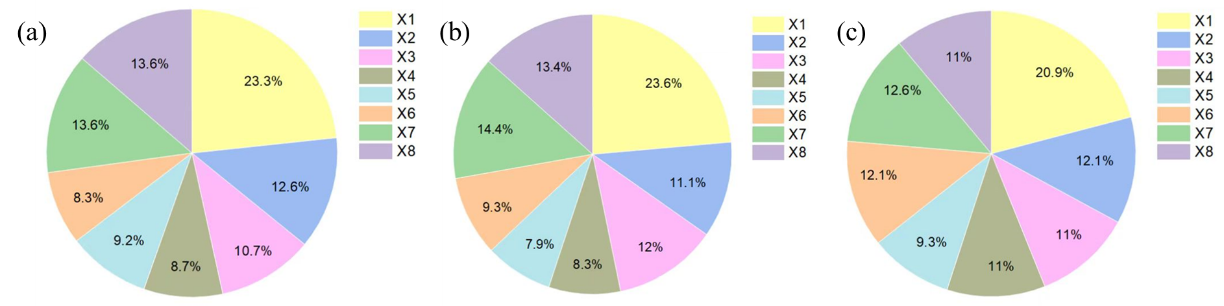
Figure S1**. Occurrences of populations of 8 properties for (a) G(H*), (b) G(C*OOH), (c) G(O*CHO) prediction by genetic algorithm (GA) method.


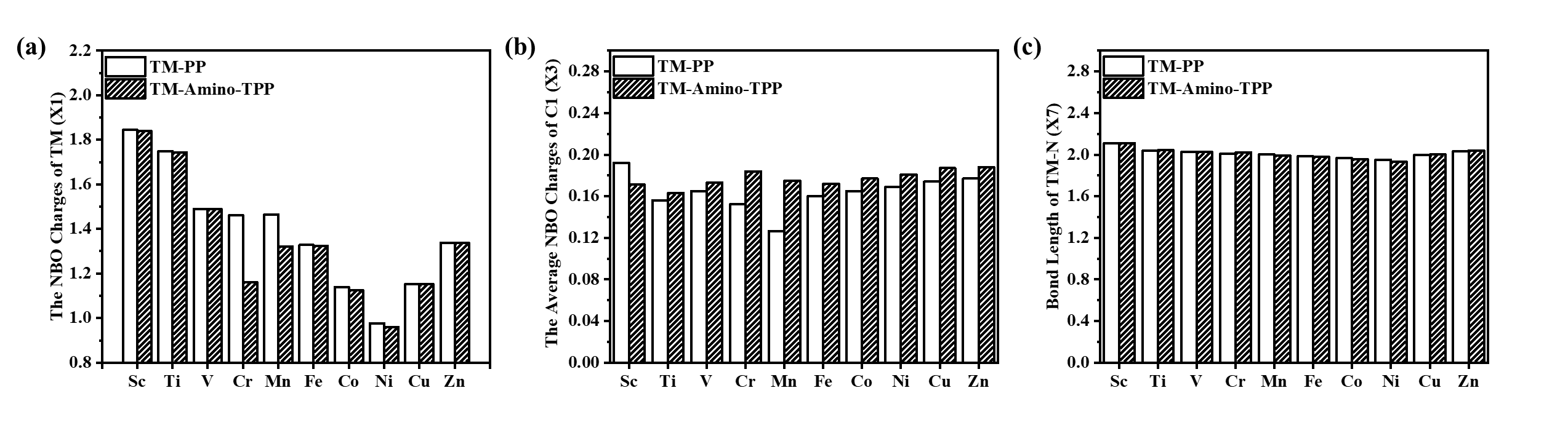
**Figure S2**. The plot of the descriptors (a) X1, (b) X3 and (c) X7 values of TM-PPs versus TM-Amino-TPPs.
