# Supplementary material for: Quantitative Structure–Electrochemistry Relationship (QSER) Studies on Metal–Amino–Porphyrins for the Rational Design of CO2 Reduction Catalysts
Source: Molecules. 2023 Mar 30;28(7):3105. doi: 10.3390/molecules28073105 (PMC10096077; doi:10.3390/molecules28073105)

Supplementary Materials

Quantitative Structure–electrochemistry Relationship (QSER) Studies on Metal-Amino-Porphyrins for Rational Design of CO_2 Reduction Catalysts

No	Figure/Table	Page
**1.**	**Table S1.** The obtained QSER equations to predict the Gibbs free energy values of H* (Y1) for TM-PPs.	S3
**2.**	**Table S2**. The obtained QSER equations to predict the Gibbs free energy values of C*OOH (Y2) for TM-PPs.	S4
**3.**	**Table S3**. The obtained QSER equations to predict the Gibbs free energy values of O*CHO (Y3) for TM-PPs.	S5
**4.**	**Table S4**. The predicted Gibbs free energy values of H*, C*OOH and O*CHO by QSER models for TM-Amino-TPPs.	S6
**5.**	**Table S5**. The selected 3 properties values of newly designed transitional metal porphyrin complexes.	S7
**6.**	**Figure S1**. Occurrences of populations of 8 properties for (a) G(H*), (b) G(C*OOH), (c) G(O*CHO) prediction by genetic algorithm (GA) method.	S8
**7.**	**Figure S2**. The plot of the descriptors (a) X1, (b) X3 and (c) X7 values of TM-PPs versus TM-Amino-TPPs.	S8

Table S1. The obtained QSER equations to predict the Gibbs free energy values of H* (Y1) for TM-PPs.

No	Equation	F-value	R^2	R^2(CV)	RSS	RMSE
Eq. 1	Y1 = - 5.994 * X1- 14.18 * X3+ 35.934 * X7 - 60.834	9.089	0.820	0.603	0.754	0.275
Eq. 1A	Y1 = 383.311 * ramp (X3 - 0.183)<br>+ 29.037 * ramp (X7 - 1.953)<br>+ 33.1439 * ramp (2.069 - X7)<br>- 3.440	34.273	0.945	0.832	0.231	0.152
Eq. 1B	Y1 = 10.271 * ramp (X1 - 1.019)	32.113	0.941	0.804	0.245	0.157

	+ 401.503 * ramp(X3 - 0.184)<br>+ 11.016 * ramp(1.733 - X1)<br>- 7.352					
Eq. 1C	Y1 = 381.573 * ramp (X3 - 0.183)<br>+ 35.183 * ramp (X7 - 1.949)<br>+ 39.346 * ramp (2.075 - X7)<br>- 4.532	34.286	0.945	0.835	0.230	0.152
Eq.1D	Y1 = 16.1652 * ramp (X7 - 1.957)<br>+ 3.189 * ramp (1.706 - X1)<br>- 1.345	3.029	0.464	0.000	2.241	0.871

Table S2. The obtained QSER equations to predict the Gibbs free energy values of C*OOH (Y2) for TM-PPs.

No	Equation	F-value	R^2	R^2(CV)	RSS	RMSE
Eq. 2	Y2 = - 5.850 * X1 - 17.701* X3 + 33.585 * X7- 56.143	12.238	0.860	0.683	0.471	0.217
Eq. 2A	Y2 = 11.025* ramp (X1 - 1.011) + 367.618 * ramp(X3 - 0.184) + 11.838 * ramp(1.768 - X1) - 8.726	44.384	0.957	0.924	0.144	0.120
Eq. 2B	Y2 = 10.271 * ramp (X1 - 1.019) + 401.503 * ramp(X3 - 0.184) + 11.016 * ramp(1.733 - X1) - 7.352	44.372	0.957	0.923	0.145	0.1120
Eq. 2C	Y2 = 332.971 * ramp (X3 - 0.183) + 41.718 * ramp (X7 - 1.946) + 46.059 * ramp (2.087 - X7) - 6.345	38.441	0.951	0.916	0.166	0.129
Eq. 2D	Y2 = 14.860 * ramp (X7 - 1.957) + 3.002 * ramp (1.799 - X1) - 1.851	3.856	0.524	0.047	1.594	0.527

Table S3. The obtained QSER equations to predict the Gibbs free energy values of O*CHO (Y3) for TM-PPs.

No	Equation	F-value	R^2	R^2(CV)	RSS	RMSE
Eq. 3	Y3 = - 4.122 * X1 + 11.431 * X7 - 17.236	9.280	0.726	0.574	1.692	0.404
Eq. 3A	Y3 = 2354.560 * ramp (X3 - 0.177) - 2043.245 * ramp (X3 - 0.176) + 25.754 * ramp (2.033 - X7) - 1.010	42.490	0.955	0.907	0.278	0.167
Eq. 3B	Y3 = 244.003 * ramp (X3 - 0.184) + 2.692 * ramp(1.635 - X1) - 1.031	14.212	0.802	0.670	1.221	0.349
Eq. 3C	Y3 = - 832.681 * ramp (X3 - 0.176) + 1141.851 * ramp (X3 - 0.178) + 25.710 * ramp (2.033 - X7) - 1.009	42.476	0.955	0.907	0.278	0.167
Eq. 3D	Y3 =- 25.354* ramp (X1 - 1.433) + 31.147 * ramp (X1 - 1.547) + 0.438	9.735	0.736	0.641	1.634	0.483

Table S4. The predicted Gibbs free energy values of H*, C*OOH and O*CHO by QSER models for TM-Amino-TPPs.

TM-Amino-TPPs	Predicted G(H*) values/eV		Predicted G(C*OOH) values/eV		Predicted G(O*CHO) values/eV	
	Eq. 1	Eq. 1A	Eq. 2	Eq. 2A	Eq. 3	Eq. 3A
Sc	1.466	1.075	0.869	0.395	-0.72	-1.01
Ti	-0.18	0.066	-0.611	-0.373	-1.068	-1.01
V	0.56	0.14	0.099	-0.166	-0.222	-0.812
Cr	2.191	0.459	1.658	0.1	1.068	-0.264
Mn	0.376	0.271	-0.04	-0.031	0.096	0.012
Fe	-0.09	0.328	-0.463	-0.032	-0.068	0.373
Co	0.229	0.419	-0.138	0.128	0.493	-0.15
Ni	0.233	1.15	-0.11	0.834	0.88	1.094
Cu	1.519	1.687	1.017	1.179	0.892	1.159
Zn	1.719	1.918	1.154	1.395	0.549	0.654

Table S5. The selected 3 properties values of newly designed transitional metal porphyrin complexes.

TM-Amino-TPP	X1<br>NBO charges of TM	X3<br>The average NBO charges of C_a	X7<br>The average bond length of TM-N_a and TM-N_b
Sc	1.839	0.171	2.108
Ti	1.743	0.163	2.043
V	1.488	0.173	2.025
Cr	1.161	0.184	2.020
Mn	1.322	0.175	1.993
Fe	1.323	0.172	1.979
Co	1.126	0.177	1.957
Ni	0.960	0.181	1.931
Cu	1.151	0.187	2.001
Zn	1.337	0.188	2.038

Figure S1. Occurrences of populations of 8 properties for (a) G(H*), (b) G(C*OOH), (c) G(O*CHO) prediction by genetic algorithm (GA) method.

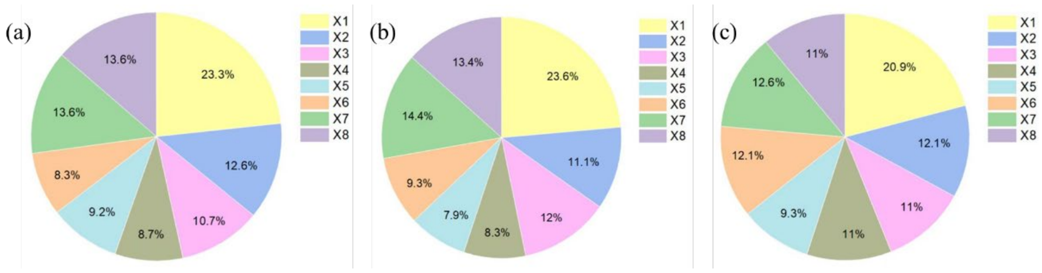

Figure S2. The plot of the descriptors (a) X1, (b) X3 and (c) X7 values of TM-PPs versus TM-Amino-TPPs.

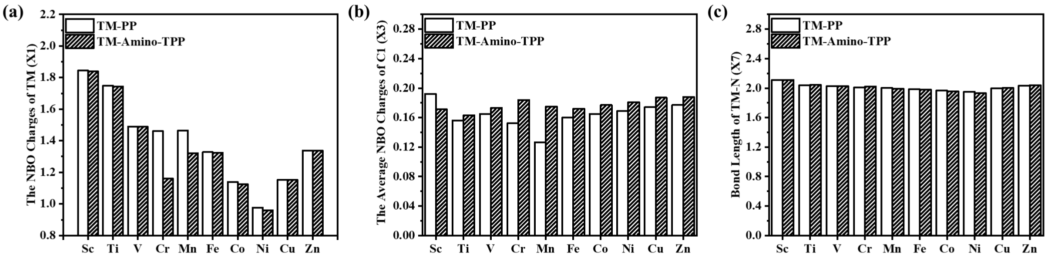

Supplement: Supplementary file 1 [file molecules-28-03105-s001.zip › molecules-2299533-supplementary.pdf]
